# Surgical Management of Recurrent Tracheocarotid Fistula following Endovascular Stent Placement

**DOI:** 10.1155/2015/547248

**Published:** 2015-11-29

**Authors:** Jeffrey T. Steitz, Zachary J. Cappello, Ziad Katrib, Paul A. Tennant

**Affiliations:** University of Louisville School of Medicine, Department of Otolaryngology-Head and Neck Surgery, Louisville, KY 40202, USA

## Abstract

We report the case of a 25-year-old woman who developed a tracheocarotid fistula secondary to an infected endovascular stent placed in the right carotid artery after the patient experienced hemorrhage on her first tracheostomy change. The patient originally had the tracheostomy placed at an outside hospital in September 2014, due to prolonged intubation after a motor vehicle accident. The patient presented to the otolaryngology service with an acute tracheal hemorrhage. This necessitated a neck exploration, median sternotomy, right carotid stent removal with subclavian to carotid bypass, and sternocleidomastoid flap reconstruction. This paper addresses the epidemiology and anatomy of a tracheocarotid fistula and discusses methods to treat such a complication.

## 1. Introduction

Massive fatal hemorrhage resulting from a fistulous tract between the trachea and a major blood vessel is an uncommon but recognized complication of the tracheostomy procedure [1]. Koerte in 1879 was the first to report such a fatality in a 5-year-old patient who suffered hemorrhage from a ruptured tracheoinnominate fistula. Multiple studies have shown that the innominate artery is the vessel that is most commonly involved in this complication [2]. However, there is a variant of this phenomenon that involves the right common carotid artery. It occurs in approximately 5% of cases [2]. Proper identification and management of such a variant are important for successful outcomes in treating patients with such a complication.

Carotid artery rupture (CAR) is a catastrophic event that is associated with a neurological morbidity of 60% and a mortality of 40% [3]. carotid artery rupture usually occurs following surgery or radiation therapy for head and neck malignancies and a significant predisposing factor is infection. Occurrence of infection can complicate the management of CAR, delaying wound healing and causing tissue necrosis and blood vessel infiltration. Infected CAR is associated with a high rate of recurrent bleeding and a high risk of cerebral complications [3].

## 2. Case Report

The patient is a 25-year-old female with a history of intravenous drug abuse. Due to this, the patient developed tricuspid vegetations and subsequently underwent a median sternotomy and tricuspid valvuloplasty one year prior to our encounter.

The patient was involved in a motor vehicle accident 8 months prior to presenting to our service. She was an unrestrained driver and was thrown from the vehicle. She was subsequently intubated in the field. The patient underwent three separate intubations for neurosurgical procedures and was ultimately unable to be weaned from the ventilator at the completion of the last procedure.

A tracheostomy was subsequently performed without complication per medical records. The patient was downsized from a size 6 cuffed tracheostomy to a size 6 cuffless tracheostomy on post-operative day 5 but had the immediate development of massive hemorrhage from her tracheostomy site. The patient was taken to interventional radiology where active extravasation from the right common carotid artery was visualized and a stent was placed.

**Figure 1:**
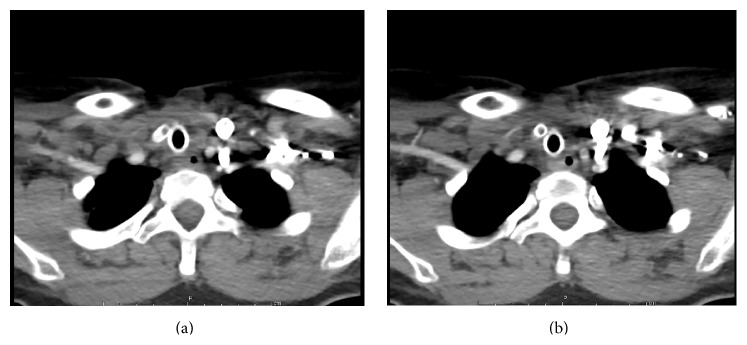
(a, b) Tracheocarotid fistula with carotid stent in place.

**Figure 2:**
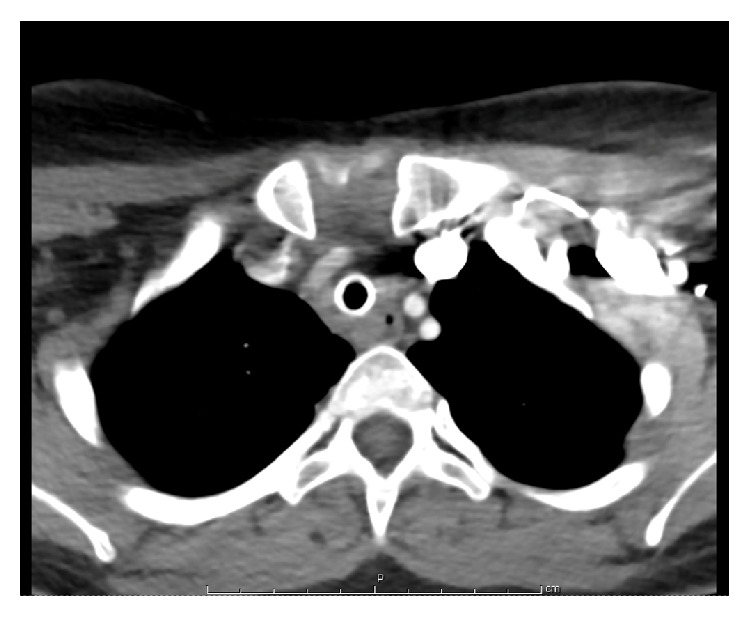
Innominate artery without fistulous tract.

Roughly 5 months later, the patient presented to our otolaryngology service with a history of two moderate volume bleeding events from her tracheostomy and a desire to be decannulated. A stroboscopy was performed, and the patient was felt to have normal vocal fold mobility with concerns for subglottic stenosis. Due to her complex medical history and known carotid stent, a CT angiogram of the head, neck, and chest was ordered (Figures 1(a), 1(b), and 2).

No extravasation was observed on CT angiogram. The right common carotid stent was noted to be 1 cm from the take-off of the common carotid artery from the innominate and was roughly 4 cm in length. The following morning, the patient presents to an outside emergency department with massive hemorrhage from her tracheostomy. The patient was subsequently transferred emergently to our University Hospital. At the time of presentation, the patient had no active bleeding. A size 8 cuffed tracheostomy tube was placed to her stoma, and the patient was taken to the operating room for a right neck exploration with concerns for a recurrent right sided tracheocarotid fistula.

**Figure 3:**
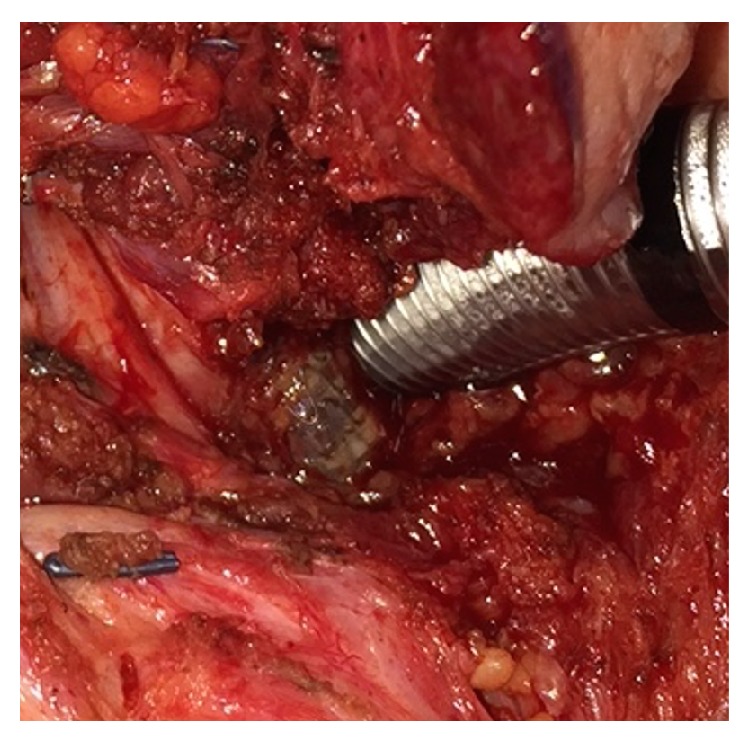
Intraoperative view of exposed carotid stent juxtaposed to trachea.

In coordination with the thoracic and vascular surgery service, the patient underwent a right neck exploration, ligation and transection of the right common carotid artery, removal of a right common carotid stent, a right carotid to subclavian end-to-side anastomosis, and a right sternocleidomastoid muscle flap to repair the right sided tracheal fistula site. The findings included an exposed stent in the common carotid artery within the tracheocarotid fistula (Figure 3) which had necrotic and purulent debris in the wound.

Postoperatively, patient was weaned from the ventilator in a matter of hours. Chest tube was removed on postoperative day three. Patient was transferred to a floor bed and passed a modified barium swallow study. She was given a regular diet on postoperative day 5 and discharged home with close follow-up. On follow-up, patient developed a mild tracheitis, successfully treated with Levofloxacin. Subsequent bronchoscopy has shown that the patient has a roughly 80% subglottic stenosis, and after the patient has healed we plan to begin attempts at dilation of her stenotic segment.

## 3. Discussion

Tracheoinnominate (TIF) and tracheocarotid fistulas (TCF) are known complications after performance of a tracheostomy. When bleeding complications occur following tracheostomy, these most commonly arise in the first several weeks; however, they may develop years following the intervention. The initial management algorithm for TIF and TCF involves immediate control of profuse hemorrhage. This entails placement and hyperinflation of cuffed tracheostomy tube, digital compression of the involved vasculature, and operative or endovascular intervention [4]. Less well elucidated is the management of hemorrhage following previous intervention. As this event is fatal in 75–80% of patients overall, and up to 100% in patients that do not undergo operative intervention, few patients are lucky enough to live for multiple carotid or innominate artery ruptures [4]. In this case the patient had previously undergone treatment with endovascular common carotid stent placement which complicated her presentation. TIF is much more frequently associated with acute arterial hemorrhage in comparison to TCF which represents approximately 5% of acute bleeding episodes [2]. Here we present the case of recurrent acute tracheostomy hemorrhage due to a tracheocarotid fistula in the presence of an infected common carotid artery stent.

In this setting, the patient presented with recurrent sentinel bleeding episodes that were self-limited and resolved with hyperinflation of the tracheostomy tube cuff. As stated above, the mortality rate of patients that do not undergo operative intervention after development of TIF/TCF approaches 100%. Despite these sentinel bleeding episodes being self-limited, this patient required further operative intervention. Neck exploration elucidated the etiology of the bleed which was an infected and exposed carotid artery stent. Infections in carotid stents are rare events themselves and only several case reports exist which detail this potentially fatal complication [5]. The rate of infection may obviously increase in the setting of tracheocarotid fistula and exposure to airflow and respiratory pathogens. In order to decrease the likelihood of any further bleeding, that exposure must be removed from the equation. As the stent was in the proximal common carotid artery, some length was gained distally up towards the carotid bifurcation which allowed for an end-to-side anastomosis to the subclavian artery. Additional options include saphenous vein graft or resection of the common carotid artery. However, in the setting of an incomplete circle of Willis (as in this case), common carotid artery sacrifice may have unintended neurologic sequelae. Ligation of the common carotid artery has been associated with major morbidity in up to 60% of patients [3]. Once the arterial bleeding is addressed, attention need be turned toward minimizing further risk of infection and fistulae formation.

Additional bolstering between the arterial repair and trachea may decrease the likelihood of further bleeding and minimize exposure of the carotid artery to airflow and respiratory pathogens as the stoma continues to heal. The sternocleidomastoid muscle (SCM) flap has been described in closures of orocutaneous, pharyngocutaneous, and tracheocutaneous fistulae as well as more complex reconstructions. The SCM flap can be harvested as a muscle flap, myocutaneous flap, myoperiosteal flap, or myosseous flap [6]. In addition to obliterating the space between the vascular repair and the trachea, reconstruction with a vascularized muscle flap also provides improved blood flow to the infected surgical bed which may improve and expedite healing [7]. Similarly, pectoralis major muscle flaps have been described in this location as well [4].

The immediate management of tracheoinnominate and tracheocarotid fistulae has been relatively well elucidated. However, without surgical intervention, mortality approaches unacceptable levels. Here we describe a unique presentation where this patient had a previous intervention for a tracheocarotid fistula that was managed with endovascular intervention. The primary goals of the intervention were to remove the infected foreign body, maintain vascularity within the common carotid artery, and minimize future risk and morbidity. This was achieved with resection and reanastomosis of the common carotid artery and a sternocleidomastoid muscle flap and has yielded a good result without further bleeding to date.

Tracheocarotid fistulae pose a complex problem with high morbidity and mortality. Recurrence of tracheocarotid fistulae after previous endovascular intervention is a difficult problem that requires multidisciplinary surgical intervention.

## References

[1] Tungekar M. F. (1999). Tracheocarotid artery fistula infected with methicillin-resistan *Staphylococcus aureus*. *The Journal of Laryngology and Otology*.

[2] Billy M. L., Snow N. J., Haug R. H. (1994). Tracheocarotid fistula with life-threatening hemorrhage: report of case. *Journal of Oral and Maxillofacial Surgery*.

[3] Liu J., Zeng Q., Huang J.-J., Hu G.-H. (2014). Management of infected carotid artery rupture. *European Archives of Oto-Rhino-Laryngology*.

[4] Ridley R. W., Zwischenberger J. B. (2006). Tracheoinnominate fistula: surgical management of an iatrogenic disaster. *Journal of Laryngology and Otology*.

[5] Son S., Choi N.-C., Choi D. S., Cho O. H. (2015). Carotid stent infection: a rare but potentially fatal complication of carotid artery stenting. *BMJ Case Reports*.

[6] Kierner A. C., Zelenka I., Gstoettner W. (2001). The sternocleidomastoid flap: its indications and limitations. *The Laryngoscope*.

[7] Losken A., Rozycki G. S., Feliciano D. V. (2000). The use of the sternocleidomastoid muscle flap in combined injuries to the esophagus and carotid artery or trachea. *Journal of Trauma—Injury, Infection and Critical Care*.

